# Multiple-to-multiple path analysis model

**DOI:** 10.1371/journal.pone.0247722

**Published:** 2021-03-04

**Authors:** Yujie Du, Junli Du, Xi Liu, Zhifa Yuan

**Affiliations:** College of Sciences, Northwest A&F University, Yangling, P. R. China; Central State University & Ohio University, UNITED STATES

## Abstract

One-to-multiple path analysis model describes the regulation mechanism of multiple independent variables to one dependent variable by dividing the correlation coefficient and the determination coefficient. How to analyse more complex regulation mechanisms of multiple independent variables to multiple dependent variables? Similarly, according to multiple-to-multiple linear regression analysis, multiple-to-multiple path analysis model was proposed in this paper and it demonstrated more complex regulation mechanisms among multiple independent variables and multiple dependent variables by dividing the generalized determination coefficient. Differently, three other types of paths were generated in multiple-to-multiple path analysis model in that the correlation among multiple dependent variables was considered. Then, the decision coefficient of each independent variable was constructed for dependent variables system, and its hypothesis testing statistics were given. Finally, the research example of the wheat breeding rules in arid area demonstrated that the multiple-to-multiple path analysis considering more correlation information can get better results.

## 1 Introduction

The regression analysis, as one of the most widely used statistical methodologies, focuses on studying the relations between dependent variables and independent variables. However, the regression analysis worries less about the correlation mechanisms that may exist among the independent variables [1]. In 1918–1921, the issue was addressed by the biological geneticist Sewall Wright through developing the path analysis method [2, 3]. Sewall Wright’s path analysis mainly emphasizes decomposing the correlation and total determination in terms of model parameters, and drawing the path diagram. The path diagram is a pictorial representation of a system of simultaneous equations, which presents the picture of the relationships that are assumed and is more clearly than the equations [4]. The concrete decomposition result is to distinguish the three types of effects: direct, indirect and total effects, which can lead to a more comprehensive understanding of the relation between variables. Usually, the indirect effects of a variable are mediated by at least one intervening variable [4]. In fact, the decomposed indirect effects quantify the regulation of variables with correlation. The quantitative expression of regulatory mechanism can make the analysis more thorough and clear. Therefore, the path analysis was later applied in multiple science research fields, such as behavioural science, social science, economics, biology, agriculture, medical science and so on [5–18]. This method seems to be more and more widely used at present.

In terms of methodology research, the path analysis was generalized to the structural equation models (SEMs) through combining the principle of factor analysis and was used to analyse the relations between multivariate blocks of data [19, 20]. The decision coefficient was constructed in the specified path analysis model with no latent variables, which included one dependent variable (as result) and multiple independent variables (as causes), based on the decomposition of total determination coefficient [21]. Here, the specific path analysis model was called one-to-multiple path analysis model with the nature of standard multiple linear regression. The decision coefficient of each independent variable equals to the sum of its direct determination and the correlation indirect determination with the other independent variables. The decision coefficient can express the magnitude and direction of each independent variable influencing the variation of dependent variable. Still further, the importance of each independent variable for dependent variable can be ranked according to the decision coefficient result, which shows that the decision coefficient has the significance of making decisions. Subsequently, the statistical test of the decision coefficient was proposed [22]. The decision coefficient improves the one-to-multiple path analysis model to a certain extent. Later, the one-to-multiple path analysis model was applied in the lint yield of upland cotton research and the KEGG gene pathway regulation mechanisms research [23–25].

However, the causal system including multiple independent variables (as “causes”) and multiple dependent variables (as “results”) are often encountered in practice research. For instance, the different pathways contain the same genes in the KEGG pathway, which demonstrated that the same genes can lead to the different gene functions. Here, multiple identical genes and multiple different gene functions constitute a multiple-to-multiple system. Analysis of the regulatory relationship between genes and gene functions is helpful to the modification and change of gene structure. Similar to this, in breeding field, multiple biological shapes to multiple yield indicators also constitute a multiple-to -multiple system. Determining the importance of multiple biological shapes to multiple yield indicators is helpful to improve the yield and quality of crops. It is assumed that such a causal system does not contain latent variables. Then, the one-to-multiple path analysis model can be used to analyse the importance of each independent variable to one dependent variable and the regulations among multiple independent variables. But, it is frustrating that the results of multiple single one-to-multiple path analysis are often contradictory, so that decision makers feel confused when making decisions. Therefore, it is urgent to find a more suitable model to provide more clear decision-making suggestions for decision-makers in such a more complex system.

In this paper, we attempt to propose the multiple-to-multiple path analysis model according to the multiple-to-multiple linear regression analysis, including multiple independent variables and multiple dependent variables and no latent variables. This model considers the correlation among multiple dependent variables caused by multiple common independent variables on the basis of one-to-multiple path analysis model. The other three types of paths generated besides the two types of paths in one-to-multiple path analysis model. The decomposition of the generalized determination coefficient showed the regulation mechanisms among the multiple independent variables and multiple dependent variables along these five types of paths. And the decision coefficient of each independent variable was used to judge its importance for all dependent variables system. Finally, the effectiveness of the model was verified by an example of the wheat breeding rules in arid area.

## 2 Method

### 2.1 Equations and models

The multiple-to-multiple linear regression model is the basis of the multiple-to-multiple path analysis, so it was introduced firstly. Define the following assumptions: the dependent variable of linear regression is *Y* = (*Y*_1_,*Y*_2_,⋯,*Y*_*p*_)^*T*^ and the independent variable is *X* = (*X*_1_,*X*_2_,⋯,*X*_*m*_)^*T*^. Suppose the joint distribution of $\underset{m\times 1}{X}$ and $\underset{p\times 1}{Y}$ is:

$$\left[\begin{array}{l} X\\ Y \end{array}\right]∼N_{m+p}\left(\left[\begin{array}{l} \mu_{x}\\ \mu_{y} \end{array}\right],\left[\begin{array}{l} \sum {}_{x}\;\sum {}_{xy}\\ \sum {}_{yx}\;\sum {}_{y} \end{array}\right]\right)=N_{m+p}\left(\mu ,\sum \right)$$ (1)

∑_*xy*_≠0, when both have been normalized, the joint distribution above becomes

$$\left[\begin{array}{l} X\\ Y \end{array}\right]∼N_{m+p}\left(\left[\begin{array}{l} 0\\ 0 \end{array}\right],\left[\begin{array}{ll} \rho_{x} & \rho_{xy}\\ \rho_{yx} & \rho_{y} \end{array}\right]\right)=N_{m+p}\left(0,\rho \right)$$ (2)

Among them, *ρ*_*x*_, *ρ*_*xy*_ and *ρ*_*y*_ are the correlation arrays of *X*,*X* and *Y*,*Y* respectively. *ρ* is the correlation matrix of [*X*^*T*^,*Y*^*T*^]^*T*^. Under the above assumption, the normalized multiple-to-multiple linear regression model is:

$$\begin{array}{l} \left[\begin{array}{l} Y_{i1}\\ Y_{i2}\\ \vdots \\ Y_{ip} \end{array}\right]={\left[\begin{array}{ll} \beta_{11}^{*} & \beta_{12}^{*}\cdots \beta_{1p}^{*}\\ \beta_{21}^{*} & \beta_{22}^{*}\cdots \beta_{2p}^{*}\\ \vdots & \vdots \ddots \vdots \\ \beta_{m1}^{*} & \beta_{m2}^{*}\cdots \beta_{mp}^{*} \end{array}\right]}^{T}\left[\begin{array}{l} x_{i1}\\ x_{i2}\\ \vdots \\ x_{im} \end{array}\right]+\left[\begin{array}{l} \varepsilon_{i1}\\ \varepsilon_{i2}\\ \vdots \\ \varepsilon_{ip} \end{array}\right]=\left[\begin{array}{l} \beta_{1}^{*}{}^{T}x_{i}\\ \beta_{2}^{*}{}^{T}x_{i}\\ \vdots \\ \beta_{p}^{*}{}^{T}x_{i} \end{array}\right]+\left[\begin{array}{l} \varepsilon_{i1}\\ \varepsilon_{i2}\\ \vdots \\ \varepsilon_{ip} \end{array}\right]\\ \Rightarrow Y_{i}=\beta^{*T}x_{i}+\varepsilon_{i}\Rightarrow Y=\beta^{*T}X+\varepsilon \end{array}$$ (3)

In (3), *Y*_*i*_ = [*Y*_*i*1_,*Y*_*i*2_,⋯,*Y*_*ip*_]^*T*^, *x*_*i*_ = [*x*_*i*1_,*x*_*i*2_,⋯,*x*_*im*_]^*T*^, β* is the regression parameter of the model, *i* = 1,2,⋯,*n*. Let *n* be the number of observations. We assumed that *ε*~*N*_*p*_(0,∑_*e*_) is the regression residual and has nothing to do with the value of *X*.

### 2.2 Regression hypothesis testing

Path analysis can only be carried out when the standardized regression equation is significant. Therefore, we need to perform the following four types of hypothesis tests for regression analysis before path analysis.

#### 2.2.1 Hypothesis testing of generalized complex correlation coefficient *r*_*xy*_

In multiple-to-multiple standardized linear regression equations, the joint distribution of *X* and *Y* is showed as formula (2), then the generalized determination coefficient is defined as [26]:

$$R^{2}=1-v_{xy}=1-\frac{\left|R\right|}{\left|R_{xx}\right|\left|R_{yy}\right|}=1-\left|I_{p}-B\right|\approx tr\left(B\right)-\sum_{t\neq l}\lambda_{t}^{2}\cdot \lambda_{l}^{2}$$ (4)

In Eq (4), *v*_*xy*_ is the likelihood ratio statistics for testing independence of X and Y. And $R_{xx}={\hat{\rho }}_{x}$, $R_{yy}={\hat{\rho }}_{y}$, *R* in |*R*| is the correlation matrix of X and Y. $B=\sum {}_{y}^{-1}\sum {}_{yx}\sum {}_{x}^{-1}\sum {}_{xy}=R_{yy}^{-1}U$ is the sample linear correlation matrix of *X* and *Y*, *U* is the regression square sum matrix. $\lambda_{t}^{2}$ and $\lambda_{l}^{2}$ are non-zero characteristic roots of B. $r_{xy}=R_{\left(x_{1}x_{2}\cdots x_{m}\right)\left(y_{1}y_{2}\cdots y_{p}\right)}=\sqrt{R^{2}}$ is the generalized complex correlation coefficient of *X* and *Y*. The invalid assumption of *r*_*xy*_ is *H*_0_:∑_*xy*_ = 0.When *p*>2 and *m*>2, we can use Bartlett’s approximate chi-square test:

$$V=-\left(n-1-\frac{p+m+1}{2}\right)\ln v_{xy}∼\chi^{2}\left(pm\right)$$ (5)

#### 2.2.2 Hypothesis testing of regression equation ${\hat{y}}_{\alpha }=b_{\alpha }^{*T}x\left(\alpha =1,2\cdots ,p\right)$

The invalid hypothesis is $H_{0}:\beta_{\alpha }^{*}=0$ and the corresponding *F* test statistic is:

$$F=\frac{R_{\left(\alpha \right)}^{2}/m}{\left(1-R_{\left(\alpha \right)}^{2}\right)/\left(n-m-1\right)}∼F\left(m,n-m-1\right)$$ (6)

$R_{\left(\alpha \right)}^{2}$ is the determination coefficient of *X* to *Y*_*α*_

#### 2.2.3 Hypothesis testing of components $b_{j\alpha }^{*}$ in $b_{\alpha }^{*}$

The invalid hypothesis is $H_{0j\alpha }:\beta_{j\alpha }^{*}=0$ and the t test statistic is

$$t_{j\alpha }=\frac{b_{j\alpha }^{*}}{\sqrt{c_{jj}{\hat{\sigma }}_{\alpha }^{2}}}∼t\left(n-m-1\right)$$ (7)

In (7), ${\hat{\sum }}_{e}=\frac{Q_{e}}{n-m-1}$, ${\hat{\sigma }}_{\alpha }^{2}$ is the α-th element on the main diagonal of ${\hat{\sum }}_{e}$. *c*_*jj*_ is the *j*-th element on the main diagonal of $R_{xx}^{-1}$.

#### 2.2.4 Hypothesis testing of $b_{x_{j}y}^{*}$ [27]

The invalid hypothesis is $H_{0}:{\left(\beta_{j1}^{*},\beta_{j2}^{*},\beta_{j3}^{*}\right)}^{T}=0$. The *F* test statistic is:

$$F=\left(n-m-2\right)\times \frac{1-\sqrt{\Lambda_{H_{0j}}}}{\sqrt{\Lambda_{H_{0j}}}}∼F\left[2,2\left(n-m-2\right)\right]$$ (8)

In (8), $\Lambda_{H_{0j}}=\frac{\left|Q_{e}\right|}{\left|Q_{H_{0j}}{}_{e}\right|}$. After the above four hypothesis tests, if the standardized multiple linear regression equation is significant, it is meaningful to perform path analysis.

### 2.3 Path analysis of $Y_{\alpha }=\beta_{\alpha }^{*T}x+\varepsilon_{\alpha }\left(\alpha =1,2,\cdots ,p\right)$

The first step of multiple-to-multiple path analysis is to conduct one-to-multiple path analysis for each dependent variable and all independent variables. According to the established multiple-to-multiple linear regression equation, the path analysis model is performed. The correlation coefficient $r_{jy_{\alpha }}$ of each dependent variable *Y*_*α*_(*α* = 1,⋯,*p*) and all independent variables *X* = (*X*_1_,*X*_2_,⋯,*X*_*m*_)^*T*^ and their determination coefficient $R_{\left(\alpha \right)}^{2}$ were divided following completely the previous one-to-multiple path analysis model on the basis of standardized linear regression equation [25]. Still further, the decision coefficient was constructed using the existing method [21]. According to the theoretical study of multiple linear regression analysis, the system of regular equations *R*_*xx*_b* = *R*_*xy*_ about the least squares estimation of β* can be rewritten as:

$$R_{xx}{\left(b_{1}^{*},b_{2}^{*},\cdots ,b_{p}^{*}\right)}^{T}=\left(R_{xy_{1}},R_{xy_{2}},\cdots ,R_{xy_{p}}\right)=R_{xy}$$

So

$$R_{xx}b_{\alpha }^{*}=R_{xy_{\alpha }},\alpha =1,2,\cdots ,p$$ (9)

**Fig 1:**
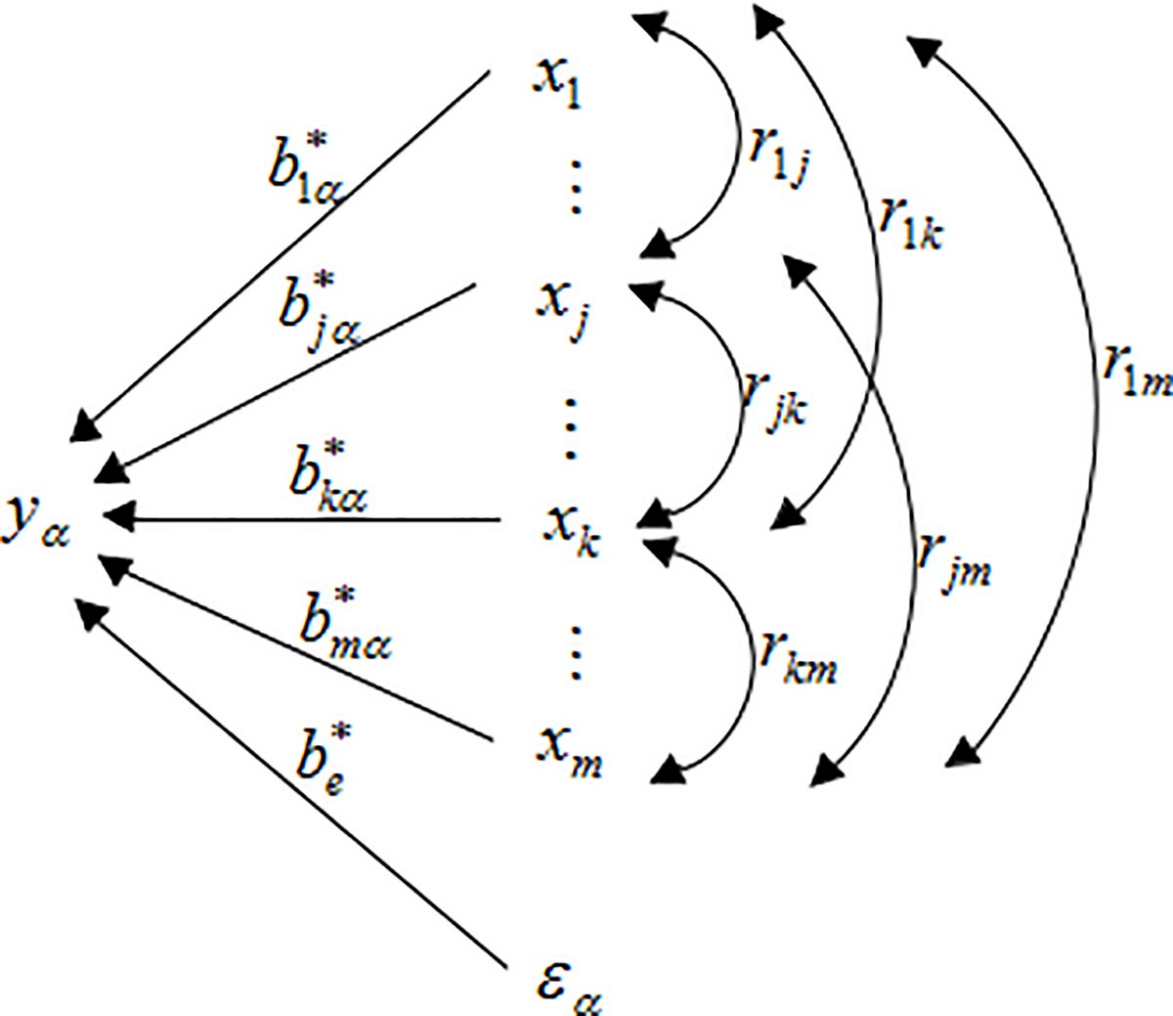
One-to-multiple path analysis diagram.

In Eq (9), $R_{xx}={\hat{\rho }}_{x}$, $R_{xy_{\alpha }}={\hat{\rho }}_{xy_{\alpha }}$. The specific path diagram of the one-to-multiple path analysis model is shown in Fig 1.

#### 2.3.1 The division and path of $r_{jy_{\alpha }}$.

$$\begin{array}{l} r_{jy_{\alpha }}=b_{j\alpha }^{*}+\sum_{k\neq j}\underset{x_{j}↔x_{k}\to y_{\alpha }}{r_{jk}b_{k\alpha }^{*}}\\ j=1,2,\cdots ,m;\;k=1,2,\cdots ,m;\alpha =1,2,\cdots ,p;\\ \underset{\varepsilon_{\alpha }\to y_{\alpha }}{b_{e\alpha }^{*}}=\sqrt{1-R_{\left(\alpha \right)}^{2}} \end{array}$$ (10)

Obviously, the correlation efficient $r_{jy_{\alpha }}$ was divided into *m* terms. There are two types for this *m* term: $b_{j\alpha }^{*}$ is formed by the path *y*_*α*_←*x*_*j*_, so $b_{j\alpha }^{*}$ is called the direct effect of *x*_*j*_ on *y*_*α*_; and $r_{jk}b_{k\alpha }^{*}\left(k\neq j\right)$ is formed by *x*_*j*_↔*x*_*k*_→*y*_*α*_, which is the effect of *x*_*j*_ on *y*_*α*_ through the correlation with *x*_*k*_ and called the indirect effect. Its magnitude can be obtained by multiplying the path coefficients $b_{k\alpha }^{*}$ by the correlation coefficient *r*_*jk*_, including *m-*1 items. Finally, $r_{jy_{\alpha }}$ is the total effect of *x*_*j*_ on *y*_*α*_, which is the sum of the direct effect and all the indirect effects.

2.3.2 The division and path of $R_{\left(\alpha \right)}^{2}$.

$$\begin{array}{l} \underset{y_{\alpha }\leftarrow x\to y_{\alpha }}{R_{\left(\alpha \right)}^{2}}=b_{\alpha }^{*T}R_{xx}b_{\alpha }^{*}=\sum_{j=1}^{m}\underset{y_{\alpha }\leftarrow x\to y_{\alpha }}{b_{j\alpha }^{*2}}+\sum_{j=1}^{m-1}2\underset{y_{\alpha }\leftarrow x_{j}↔x_{k}\to y_{\alpha }}{b_{j\alpha }^{*}r_{jk}b_{k\alpha }^{*}}\\ \;=\sum_{j=1}^{m}\underset{y_{\alpha }\leftarrow x\to y_{\alpha }}{R_{j\left(\alpha \right)}^{*2}}+\sum_{k>j}\underset{y_{\alpha }\leftarrow x_{j}↔x_{k}\to y_{\alpha }}{R_{jk\left(\alpha \right)}^{*}} \end{array}$$ (11)

Among (11): $R_{\left(\alpha \right)}^{2}$ is the total coefficient of determination of *X* for *Y*_*α*_. $R_{j\left(\alpha \right)}^{*2}=b_{j\alpha }^{*2}$ and its corresponding path is *y*_*α*_←*x*→*y*_*α*_. It is called the direct determination coefficient of *x*_*j*_ to *y*_*α*_.The corresponding path of $R_{jk\left(\alpha \right)}^{*}=2b_{j\alpha }^{*}r_{jk}b_{k\alpha }^{*}$ is *y*_*α*_←*x*_*j*_↔*x*_*k*_→*y*_*α*_. It is called the correlation determination coefficient of *x*_*j*_ through the correlation with *x*_*k*_(*k*≠*j*) to *y*_*α*_.

#### 2.3.3 The decision coefficient *R*_*α*(*j*)_ and hypothesis test [22]

The comprehensively determine ability of *x*_*j*_ to *y*_*α*_ can be represented by the decision coefficient based on the division of $R_{\left(\alpha \right)}^{2}$. Its specific expression and hypothesis test are:

$$\begin{array}{l} R_{\alpha \left(j\right)}=2b_{j\alpha }^{*}r_{jy_{\alpha }}-b_{j\alpha }^{*2}=\underset{y_{\alpha }\leftarrow x\to y_{\alpha }}{R_{j\left(\alpha \right)}^{*2}}+\sum_{k\neq j}\underset{y_{\alpha }\leftarrow x_{j}↔x_{k}\to y_{\alpha }}{R_{jk\left(\alpha \right)}^{*}}\\ t_{\alpha \left(j\right)}=\frac{R_{\alpha \left(j\right)}}{S_{R_{\alpha \left(j\right)}}}=\frac{R_{\alpha \left(j\right)}}{2\left|r_{jy_{\alpha }}-b_{j\alpha }^{*}\right|\sqrt{\frac{c_{jj}\left(1-R_{\left(\alpha \right)}^{*2}\right)}{n-m-1}}}∼t\left(n-m-1\right)\\ j=1,2,\cdots ,m;\alpha =1,2,\cdots ,p \end{array}$$ (12)

The definition indicates that *R*_*α*(*j*)_ equals to the sum of the direct determination coefficient $R_{j\left(\alpha \right)}^{*2}=b_{j\left(\alpha \right)}^{*2}$ and the correlation determination coefficient $R_{jk\left(\alpha \right)}^{*}=2b_{j\alpha }^{*}r_{jk}b_{k\alpha }^{*}\left(k\neq j\right)$. In fact, the decision coefficient is the sum of all determination coefficients related to *x*_*j*_. The decision coefficient was used to determine the main decision variables and restrictive variables affecting *Y*_*α*_.

### 2.4 Multiple-to-multiple path analysis central theorem

**Fig 2:**
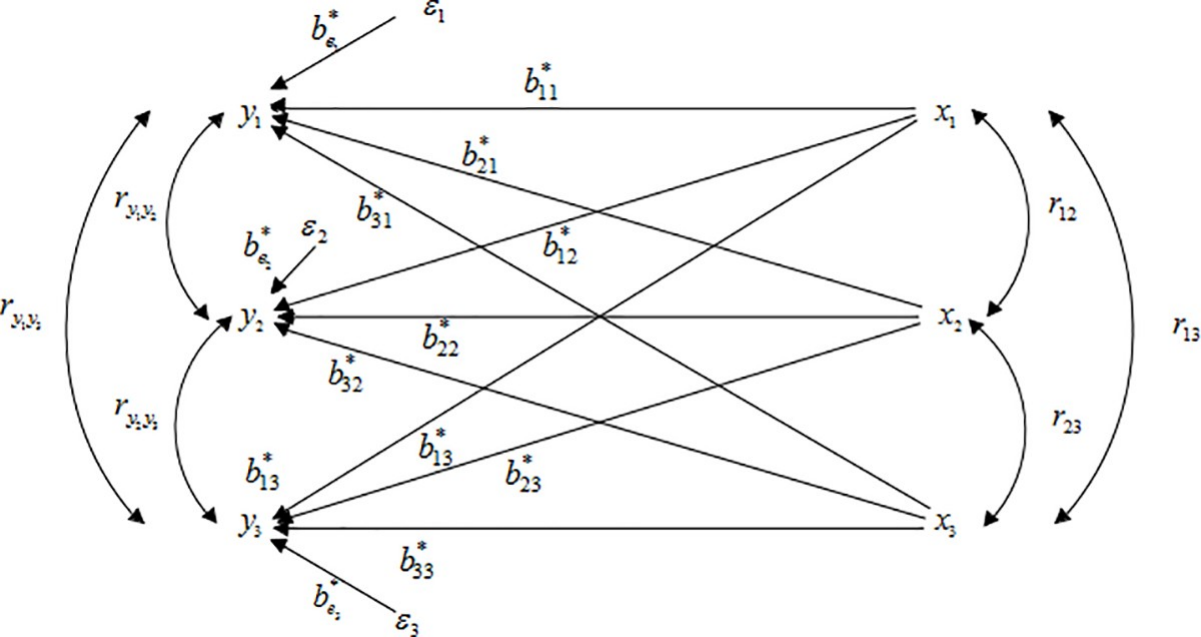
Multiple-to-multiple path analysis diagram.

The second step is to conduct multiple-to-multiple path analysis. And the innovation is that the correlation between *Y* caused by the common cause *X* is considered and three other types of paths are generated. For convenience of observation, let *p* = 3, *m* = 3 as an example to make a multiple-to-multiple path analysis diagram as Fig 2. But, the theoretical analysis is based on *m* independent variables and *p* dependent variables.

The multiple-to-multiple path analysis model considered the correlation among different dependent variables compared to the one-to-multiple path analysis model. Accordingly, the central theorem of multiple-to-multiple path analysis is proposed. Based on model (3), for two different *Y*_*α*_ and *Y*_*t*_, their models are:

$$\left\{ \begin{array}{l} Y_{\alpha }=\beta_{\alpha }^{*T}X+\varepsilon_{\alpha },\varepsilon_{\alpha }=Y_{\alpha }-E\left(Y_{\alpha }|X=x\right)\\ Y_{t}=\beta_{t}^{*T}X+\varepsilon_{t},\varepsilon_{t}=Y_{t}-E\left(Y_{t}|X=x\right) \end{array}\right.$$ (13)

In (13), *ε*_*α*_ and *ε*_*t*_ are independent of each other and have nothing to do with the value of *X*. Since *Y*_*α*_, *Y*_*t*_ and *X* have been standardized, the correlation coefficients of *Y*_*α*_ and *Y*_*t*_, and the corresponding path theoretically is:

$$\begin{array}{l} \rho_{Y_{\alpha }Y_{t}}\\ =Cov\left(\beta_{\alpha }^{*T}X+\varepsilon_{\alpha },\beta_{t}^{*T}X+\varepsilon_{t}\right)=\beta_{\alpha }^{*T}Cov\left(X,X\right)\beta_{t}^{*}=\beta_{\alpha }^{*T}\rho_{x}\beta_{t}^{*}\\ =\sum_{j=1}^{m}\underset{y_{\alpha }\leftarrow x_{j}\to y_{t}}{\beta_{j\alpha }^{*T}\beta_{jt}^{*T}}+\sum_{k\neq j}\left(\underset{y_{\alpha }\leftarrow x_{j}↔x_{k}\to y_{t}}{\beta_{j\alpha }^{*}\rho_{jk}\beta_{kt}^{*}}+\underset{y_{\alpha }\leftarrow x_{k}↔x_{j}\to y_{t}}{\beta_{k\alpha }^{*}\rho_{kj}\beta_{jt}^{*}}\right)\\ =\underset{y_{\alpha \left(X\right)↔}y_{t\left(X\right)}}{\rho_{y_{\alpha \left(X\right)}y_{t\left(X\right)}}} \end{array}$$ (14)

Considering the sample case, Eq (14) is:

$$\begin{array}{l} \underset{y_{\alpha }↔y_{t}}{r_{y_{\alpha }y_{t}}}\\ =Cov\left(b_{\alpha }^{*T}x+\varepsilon_{\alpha },b_{t}^{*T}x+\varepsilon_{t}\right)=b_{\alpha }^{*T}R_{xx}b_{t}^{*}\\ =\sum_{j=1}^{m}\underset{y_{\alpha }\leftarrow x_{j}\to y_{t}}{b_{j\alpha }^{*}b_{jt}^{*}}+\sum_{k\neq j}\left(\underset{y_{\alpha }\leftarrow x_{j}↔x_{k}\to y_{t}}{b_{j\alpha }^{*}r_{jk}r_{kt}^{*}}+\underset{y_{\alpha }\leftarrow x_{k}↔x_{j}\to y_{t}}{b_{k\alpha }^{*}r_{kj}b_{jt}^{*}}\right) \end{array}$$ (15)

Among them, *j* = 1,2,⋯,*m*; *k* = 1,2,⋯,*m*; and *α* = 1,2,⋯,*p*; *t* = 1,2,⋯,*p*. *E*q (14) and Eq (15) are called the central theorem of multiple-to-multiple path analysis.

The central theorem demonstrated that $\rho_{Y_{\alpha }Y_{t}}$, $r_{y_{\alpha }y_{t}}$ equal to the sum of *m*^2^ items composite path coefficient. Wherein, the direct path *y*_*α*_←*x*_*j*_→*y*_*t*_ has *m* items. Due to the correlation among independent variables *x*_*j*_↔*x*_*k*_(*k*≠*j*), the two types of indirect paths were formed as *y*_*α*_←*x*_*j*_↔*x*_*k*_→*y*_*t*_, *y*_*α*_←*x*_*k*_↔*x*_*j*_→*y*_*t*_. And *x*_*j*_↔*x*_*k*_(*k*≠*j*) has $C_{m}^{2}=\frac{1}{2}m\left(m-1\right)$ items. So the total composite path number is $m+2\times \frac{1}{2}m\left(m-1\right)=m^{2}$ items. In addition, the central theorem also showed that three other types of paths generated when the correlation between different dependent variables *y*_*α*_ and *y*_*t*_ was considered, which was caused by the common *X*. Therefore, there are five types of paths in multiple-to-multiple path analysis, plus the two types of paths in one-to-multiple path analysis.

In fact, the correlation coefficient in the multiple-to-multiple path analysis central theorem is theoretically the regression square sum matrix U in multiple-to-multiple standardized linear regression. Under the least squares estimation, *U* can be expressed as follows [16]:

$$\begin{array}{l} \underset{Y\leftarrow x\to Y}{U}=\underset{\hat{y}\leftarrow x\to \hat{y}}{U}=b^{*T}R_{xy}=b^{*T}R_{xx}b^{*}\\ \;=\left[\begin{array}{cccc} b_{{}^{1}}^{*T}R_{xx}b_{{}^{1}}^{*} & b_{{}^{1}}^{*T}R_{xx}b_{{}^{2}}^{*} & \cdots & b_{{}^{1}}^{*T}R_{xx}b_{{}^{p}}^{*}\\ b_{{}^{2}}^{*T}R_{xx}b_{{}^{1}}^{*} & b_{{}^{2}}^{*T}R_{xx}b_{{}^{2}}^{*} & \cdots & b_{{}^{2}}^{*T}R_{xx}b_{{}^{p}}^{*}\\ \;\vdots & \vdots & & \vdots \\ b_{{}^{p}}^{*T}R_{xx}b_{{}^{1}}^{*} & b_{{}^{p}}^{*T}R_{xx}b_{{}^{2}}^{*} & \cdots & b_{{}^{p}}^{*T}R_{xx}b_{{}^{p}}^{*} \end{array}\right]\\ \;=\left[\begin{array}{cccc} \underset{{\hat{y}}_{1}\leftarrow x\to {\hat{y}}_{1}}{R_{\left(1\right)}^{2}} & \underset{{\hat{y}}_{1}\leftarrow x\to {\hat{y}}_{2}}{r_{y_{1}y_{2}\left(x\right)}} & \cdots & \underset{{\hat{y}}_{1}\leftarrow x\to {\hat{y}}_{p}}{r_{y_{1}y_{p}\left(x\right)}}\\ \underset{{\hat{y}}_{2}\leftarrow x\to {\hat{y}}_{1}}{r_{y_{2}y_{1}\left(x\right)}} & \underset{{\hat{y}}_{2}\leftarrow x\to {\hat{y}}_{2}}{R_{\left(2\right)}^{2}} & \cdots & \underset{{\hat{y}}_{2}\leftarrow x\to {\hat{y}}_{p}}{r_{y_{2}y_{p}\left(x\right)}}\\ \;\vdots & \vdots & & \vdots \\ \underset{{\hat{y}}_{p}\leftarrow x\to {\hat{y}}_{1}}{r_{y_{p}y_{1}\left(x\right)}} & \underset{{\hat{y}}_{p}\leftarrow x\to {\hat{y}}_{2}}{r_{y_{p}y_{2}\left(x\right)}} & \cdots & \underset{{\hat{y}}_{p}\leftarrow x\to {\hat{y}}_{p}}{R_{\left(p\right)}^{2}} \end{array}\right] \end{array}$$ (16)

In (16), $b_{\alpha }^{*T}R_{xx}b_{t}^{*}=r_{{\hat{y}}_{\alpha }{\hat{y}}_{t}\left(x\right)},\alpha \neq t$ is the correlation coefficient between *y*_*α*_ and *y*_*t*_ caused by the common cause *X*. Here, *U* is the determination coefficient matrix of *X* to *Y*. $R_{\left(\alpha \right)}^{2}=r_{{\hat{y}}_{\alpha }{\hat{y}}_{\alpha }\left(x\right)}^{2}$ is the coefficient of determination of *X* to *Y*_*α*_. $\sqrt{R_{\left(\alpha \right)}^{2}}=r_{y_{\alpha }\left(x_{1}x_{2}\cdots x_{m}\right)}=r_{y_{\alpha }\left(x\right)},\alpha =1,2,\cdots ,p$ is the complex correlation coefficient of *X* to *Y*_*α*_. And in statistics, $r_{Y_{\alpha }Y_{t}}$ is the correlation coefficient of *Y*_*α*_ and *Y*_*t*_, and has nothing to do with *X* in the calculation. $r_{{\hat{y}}_{\alpha }{\hat{y}}_{t}\left(\alpha \right)}$ is the determining part of *Y*_*α*_ and *Y*_*t*_ to $r_{Y_{\alpha }Y_{t}}$ due to the common cause *X*.

### 2.5 The division of *R*^2^≈*tr*(*B*) and its corresponding path

The generalized determination coefficient has been defined using formula (4) before, which was used to reflect the comprehensive determination of all independent variables to all dependent variables [26]. Because the non-zero eigenvalue $\lambda_{t}^{2}\left(t=1,2,\cdots ,k\right)$ of *B* is small and $0<\lambda_{k}^{2}\leq \lambda_{k-1}^{2}\leq \cdots \leq \lambda_{1}^{2}\leq 1$, the result of $\Sigma_{t\neq l}\lambda_{t}^{2}\lambda_{l}^{2}$ is small enough to make *R*^2^≈*tr*(*B*). In fact, *tr*(*B*) is the overestimation of *R*^2^ here. According to *R*^2^≈*tr*(*B*), the generalized determination coefficient *R*^2^ was divided as follows:

$$\begin{array}{l} R^{2}\approx tr\left(B\right)=\sum_{\alpha =1}^{p}\theta_{\alpha \alpha }\underset{y_{\alpha }\leftarrow \underset{m\times 1}{x}\to y_{\alpha }}{R_{\left(\alpha \right)}^{2}}+2\sum_{t>\alpha }\theta_{\alpha t}\underset{y_{\alpha }\leftarrow \underset{m\times 1}{x}\to y_{t}}{r_{y_{\alpha }y_{t}(x)}}\\ \;=\sum_{\alpha =1}^{p}\theta_{\alpha \alpha }\left(\sum_{j=1}^{m}\underset{y_{\alpha }\leftarrow x_{j}\to y_{\alpha }}{b_{j\alpha }^{*2}}+2\sum_{\begin{array}{l} j=1\\ k>j \end{array}}^{m-1}\underset{y_{\alpha }\leftarrow x_{j}↔x_{k}\to y_{\alpha }}{b_{j\alpha }^{*}r_{jk}b_{k\alpha }^{*}}\right)\\ \;+\sum_{t>\alpha }\theta_{\alpha t}\left[\sum_{j=1}^{m}2\underset{y_{\alpha }\leftarrow x_{j}\to y_{t}}{b_{j\alpha }^{*}b_{jt}^{*}}+\sum_{k\neq j}\left(2\underset{y_{\alpha }\leftarrow x_{j}↔x_{k}\to y_{t}}{b_{j\alpha }^{*}r_{jk}b_{kt}^{*}}+2\underset{y_{\alpha }\leftarrow x_{k}↔x_{j}\to y_{t}}{b_{k\alpha }^{*}r_{kj}b_{jt}^{*}}\right)\right]\\ \;=\sum_{j=1}^{m}\sum_{\alpha =1}^{p}\theta_{\alpha \alpha }\underset{y_{\alpha }\leftarrow x_{j}\to y_{\alpha }}{R_{j\left(\alpha \right)}^{2}}+\sum_{k<j}^{}\sum_{\alpha =1}^{p}\theta_{\alpha \alpha }\underset{y_{\alpha }\leftarrow x_{j}↔x_{k}\to y_{\alpha }}{R_{jk\left(\alpha \right)}^{}}\\ \;+\sum_{t>\alpha }\theta_{\alpha t}\left[\sum_{j=1}^{m}\underset{y_{\alpha }\leftarrow x_{j}\to y_{t}}{R_{j\left(\alpha t\right)}^{}}+\sum_{k\neq j}\left(\underset{y_{\alpha }\leftarrow x_{j}↔x_{k}\to y_{t}}{R_{jk\left(\alpha t\right)}^{}}+\underset{y_{\alpha }\leftarrow x_{k}↔x_{j}\to y_{t}}{R_{kj\left(\alpha t\right)}^{}}\right)\right] \end{array}$$ (17)

Among (17), *θ*_*αt*_ is the element in matrix $R_{{}_{yy}}^{-1}$. $R_{{}_{j(\alpha )}}^{2}=b_{j\alpha }^{*2}$ is the direct determination coefficient of *x*_*j*_ on *y*_*α*_, and the effect path is *y*_*α*_←*x*_*j*_→*y*_*α*_, *j* = 1,2,⋯,*m*; *α* = 1,2,⋯,*p*. $R_{jk\left(\alpha \right)}=2b_{j\alpha }^{*}r_{jk}b_{k\alpha }^{*}$ is the indirect determination coefficient of *x*_*j*_ and *x*_*k*_ on *y*_*α*_, the effect path is *y*_*α*_←*x*_*j*_↔*x*_*k*_→*y*_*α*_, *jk* has $\frac{1}{2}m\left(m-1\right)$ items. $R_{j\left(\alpha t\right)}=2b_{j\alpha }^{*}b_{jt}^{*}$ is the direct determination coefficient of *x*_*j*_ on *y*_*α*_ and *y*_*t*_, which is caused by the correlation of *y*_*α*_ and *y*_*t*_ because of the common cause *x*_*j*_. The effect path is *y*_*α*_←*x*_*j*_→*y*_*t*_, *j* = 1,2,⋯,*m*, *αt* has $\frac{1}{2}p\left(p-1\right)$ items. $R_{jk\left(\alpha t\right)}=2b_{j\alpha }^{*}r_{jk}b_{kt}^{*}$ is the indirect determination coefficient of *x*_*j*_ and *x*_*k*_ on *y*_*α*_ and *y*_*t*_. The effect path is *y*_*α*_←*x*_*j*_↔*x*_*k*_→*y*_*t*_. When *α*<*t*, *y*_*α*_ and *y*_*t*_ have $\frac{1}{2}p\left(p-1\right)$ items; when *j*≠*k*, *jk* has $\frac{1}{2}m\left(m-1\right)$ items. $R_{kj\left(\alpha t\right)}=2b_{k\alpha }^{*}r_{kj}b_{jt}^{*}$ is the indirect determination coefficient of *x*_*j*_ and *x*_*k*_ on *y*_*t*_ and *y*_*α*_. The effect path is *y*_*α*_←*x*_*k*_↔*x*_*j*_→*y*_*t*_, when *α*<*t*, *y*_*α*_ and *y*_*t*_ have $\frac{1}{2}p\left(p-1\right)$ items; when *j*≠*k*, *kj* has $\frac{1}{2}m\left(m-1\right)$ items. Therefore, the total number of items divided is:

$$\begin{array}{l} p\left(m+\frac{1}{2}m\left(m-1\right)\right)+\frac{1}{2}p\left(p-1\right)\left(m+\frac{1}{2}m\left(m-1\right)\right)\\ =\frac{pm}{4}\left(m+1\right)\left(p+1\right) \end{array}$$

Formula (17) demonstrates that the generalized determination coefficient *R*^2^ was divided successfully along the five types of paths stated in multiple-to-multiple path analysis central theorem. The specific path vector structure is:

$$\begin{array}{l} \underset{\underset{p\times 1}{y}↔\underset{m\times 1}{x}}{R^{2}}\approx tr\left(B\right)\\ =\left(\theta_{11},\theta_{22},\cdots ,\theta_{pp}\right)\times \left(\sum_{j=1}^{m}\underset{\underset{p\times 1}{y}\leftarrow x_{j}\to \underset{p\times 1}{y}}{\left[\begin{array}{l} R_{j\left(1\right)}^{2}\\ R_{j\left(2\right)}^{2}\\ \vdots \\ R_{j\left(p\right)}^{2} \end{array}\right]}+\sum_{k>j}\underset{\underset{p\times 1}{y}\leftarrow x_{j}↔x_{k}\to \underset{p\times 1}{y}}{\left[\begin{array}{l} R_{jk\left(1\right)}^{}\\ R_{jk\left(2\right)}^{}\\ \vdots \\ R_{jk\left(p\right)}^{} \end{array}\right]}\right)\\ +\left(\theta_{12},\theta_{13},\cdots ,\theta_{\left(p-1\right)p}\right)\times \left(\sum_{j=1}^{m}\underset{\underset{\left(\alpha <t\right)}{y_{\alpha }\leftarrow x_{j}\to y_{t}}}{\left[\begin{array}{l} R_{j\left(12\right)}^{}\\ R_{j\left(13\right)}^{}\\ \vdots \\ R_{j\left(\left(p-1\right)p\right)}^{} \end{array}\right]}+\sum_{k>j}\underset{y_{\alpha }\leftarrow \left(\begin{array}{l} x_{j}↔x_{k}\\ x_{k}↔x_{j} \end{array}\right)\to y_{t}}{\left[\begin{array}{l} R_{jk\left(12\right)}^{}+R_{kj\left(12\right)}^{}\\ R_{jk\left(13\right)}^{}+R_{kj\left(13\right)}^{}\\ \vdots \\ R_{jk\left(\left(p-1\right)p\right)}^{}+R_{kj\left(\left(p-1\right)p\right)}^{} \end{array}\right]}\right) \end{array}$$ (18)

### 2.6 The generalized decision coefficient *R*_*y*(*j*)_

#### 2.6.1 The definition of *R*_*y*(*j*)_

In order to describe the comprehensive decision-making ability of *x*_*j*_ to *Y*, the generalized decision coefficient *R*_*y*(*j*)_ was defined as follows:

$$\begin{array}{l} R_{y\left(j\right)}=\left(\theta_{11},\theta_{22},\cdots ,\theta_{pp}\right)\times \left(\underset{\underset{p\times 1}{y}\leftarrow x_{j}\to \underset{p\times 1}{y}}{\left[\begin{array}{l} R_{j\left(1\right)}^{2}\\ R_{j\left(2\right)}^{2}\\ \vdots \\ R_{j\left(p\right)}^{2} \end{array}\right]}+\sum_{k\neq j}\underset{\underset{p\times 1}{y}\leftarrow x_{j}↔x_{k}\to \underset{p\times 1}{y}}{\left[\begin{array}{l} R_{jk\left(1\right)}^{}\\ R_{jk\left(2\right)}^{}\\ \vdots \\ R_{jk\left(p\right)}^{} \end{array}\right]}\right)\\ +\left(\theta_{12},\theta_{13},\cdots ,\theta_{\left(p-1\right)p}\right)\times \left(\underset{\underset{\left(\alpha <t\right)}{y_{\alpha }\leftarrow x_{j}\to y_{t}}}{\left[\begin{array}{l} R_{j\left(12\right)}^{}\\ R_{j\left(13\right)}^{}\\ \vdots \\ R_{j\left(\left(p-1\right)p\right)}^{} \end{array}\right]}+\sum_{k>j}\underset{y_{\alpha }\leftarrow \left(\begin{array}{l} x_{j}↔x_{k}\\ x_{k}↔x_{j} \end{array}\right)\to y_{t}}{\left[\begin{array}{l} R_{jk\left(12\right)}^{}+R_{kj\left(12\right)}^{}\\ R_{jk\left(13\right)}^{}+R_{kj\left(13\right)}^{}\\ \;\vdots \\ R_{jk\left(\left(p-1\right)p\right)}^{}+R_{kj\left(\left(p-1\right)p\right)}^{} \end{array}\right]}\right)\\ =R_{y(j)Ι}+R_{y(j)\Pi },j=1,2,\cdots ,m \end{array}$$ (19)

Obviously, the generalized decision coefficient is the sum of the products of $R_{j\left(\alpha \right)}^{2}$, *R*_*jk*(*α*)_, *R*_*j*(*αt*)_ and *R*_*jk*(*αt*)_+*R*_*kj*(*αt*)_ related to *x*_*j*_ in the division and the corresponding elements in $R_{yy}^{-1}={\left(\theta_{\alpha t}\right)}_{p\times p}$ on the basis of *R*^2^≈*tr*(*B*). In (19), *R*_*y*(*j*)_ is divided into two parts: *R*_*y*(*j*)I_ and *R*_*y*(*j*)*II*_. *R*_*y*(*j*)I_ is the determination part of *x*_*j*_ and *x*_*j*_↔*x*_*k*_ to *Y*_*α*_. *R*_*y*(*j*)Π_ is the determination part of *x*_*j*_and *x*_*j*_↔*x*_*k*_ to *Y*_*α*_ and *Y*_*t*_(*α*≠*t*) due to the common *X*. In a word, the generalized decision coefficient includes not only the direct determination of *x*_*j*_ to *Y*_*α*_, *Y*_*α*_ and *Y*_*t*_(*α*≠*t*), but also the indirect determination of *x*_*j*_↔*x*_*k*_(*k*≠*j*) to *Y*_*α*_ and *Y*_*α*_ and *Y*_*t*_(*α*≠*t*). Specially, the indirect determination considers the correlation among the independent variables and the correlation among the dependent variables at the same time. Therefore, the decision coefficient *R*_*y*(*j*)_ can be used to express the comprehensive decision ability of *x*_*j*_ to *Y*.

#### 2.6.2 The hypothesis testing of *R*_*y*(*j*)_

The invalid hypothesis is *H*_0_:*E*(*R*_*y*(*j*)_) = 0 and the corresponding t test statistic is:

$$t_{j}=\frac{R_{y\left(j\right)}}{\sqrt{\sum_{\alpha =1}^{p}{\left(R_{y\left(j\right)\alpha }^{'}\right)}^{2}\frac{c_{jj}\left(1-tr\left(B\right)\right)}{n-m-1}}}\sim t\left(n-m-1\right)$$ (20)

In (20), $R_{y\left(j\right)\alpha }^{'}=\frac{\partial R_{y\left(j\right)}}{\partial b_{j\alpha }^{*}}$.

## 3 Application

### 3.1 Datasets

In order to demonstrate the effectiveness of the multiple-to-multiple path analysis, the wheat data in arid areas to explore breeding rules was selected to discuss. In detail, the wheat data included thirty-five varieties. These data were obtained in a completely randomized block test, and each sample was set with three repetitions [28]. In multiple-to-multiple path analysis, three indexes closely related to wheat yield was selected as dependent variables: panicles per plant (*y*_1_), grain number per panicle (*y*_2_) and 1000-grain weight (*y*_3_), and three other indexes were selected as independent variables: bio-mass per plant (*x*_1_), single stem grass weight (*x*_2_) and economic coefficient (*x*_3_). Here, economic coefficient refers to the ratio of economic yield to biological yield of wheat.

### 3.2 Calculation and results

Firstly, the phenotypic correlation matrix of the sample was calculated and expressed as Eq (21). The number of observations for each variable is *n* = 105.

$$\begin{array}{l} \begin{array}{l} R=\;x_{1}\;x_{2}\;x_{3}\;y_{1}\;y_{2}\;y_{3} \end{array}\\ \begin{array}{c} \;x_{1}\\ \;x_{2}\\ \;x_{3}\\ \;y_{1}\\ \;y_{2}\\ \;y_{3} \end{array}\left[\begin{array}{cccccc} 1 & 0.711 & ‐0.367 & 0.013 & 0.225 & 0.028\\ 0.711 & 1 & ‐0.418 & ‐0.477 & 0.259 & 0.238\\ ‐0.367 & ‐0.418 & 1 & ‐0.055 & 0.173 & 0.327\\ 0.013 & ‐0.477 & ‐0.055 & 1 & ‐0.255 & ‐0.383\\ 0.225 & 0.259 & 0.173 & ‐0.255 & 1 & ‐0.058\\ 0.028 & 0.238 & 0.327 & ‐0.383 & ‐0.058 & 1 \end{array}\right] \end{array}$$ (21)

Then, we establish a multiple-to-multiple standardized multiple linear regression equation and calculate the corresponding parameters, the results were written as follow:

$$\left[\begin{array}{l} {\hat{y}}_{1}\\ {\hat{y}}_{2}\\ {\hat{y}}_{3} \end{array}\right]=b^{*T}x=\left[\begin{array}{l} 0.6767x_{1}-1.0631x_{2}-0.2510x_{3}\\ 0.1323x_{1}-0.3121x_{2}-0.3520x_{3}\\ -0.2150x_{1}-0.3121x_{2}-0.4986x_{3} \end{array}\right]$$ (22)

Right after, four types of hypothesis testing based on the established standardized multiple linear regression model were conducted as follow:

1. The hypothesis testing of generalized complex correlation coefficient *r*_*xy*_.
  Likelihood ratio statistics of *X* and *Y* is *v*_*xy*_ = 0.2987, so *χ*^2^ = 121.4357**>*χ*^2^(3×3), and *R*^2^ = 1−*v*_*xy*_ = 0.7013, $r_{xy}=r_{\left(x_{1}x_{2}x_{3}\right)\left(y_{1}y_{2}y_{3}\right)}=\sqrt{R^{2}}=0.8374^{**}$. The results showed that the linear regression of *Y* to *X* was extremely significant.
2. The hypothesis testing of regression equation ${\hat{y}}_{\alpha }=b_{\alpha }^{*T}x\left(\alpha =1,2,3\right)$.
  The values of F test statistics are *F*_1_ = 37.9188**, *F*_2_ = 25.394**, *F*_3_ = 14.408**, respectively. They were all greater than *F*_0.01_(3,101) = 4.007, which showed that each standardized regression equation was extremely significant.
3. The hypothesis testing of components $b_{j\alpha }^{*}$ in $b_{\alpha }^{*}$
  The results of hypothesis testing of components $b_{j\alpha }^{*}$ in $b_{\alpha }^{*}$ were listed in **Table 1**.

**Table 1:** t test statistics value of $b_{j\alpha }^{*}$.

	*y*_1_	*y*_2_	*y*_3_
*x*_1_	6.8994**	1.0212	-1.8092
*x*_2_	-105852**	2.3527**	4.9257**
*x*_3_	-3.3060**	3.5101**	5.4202**

Among them, except *x*_1_ was not significant to *y*_2_ and *y*_3_, the others were extremely significant.

4 The hypothesis testing of $b_{x_{j}y}^{*}$

The results are *F*_1_ = 8.670**, *F*_2_ = 25.394**, *F*_3_ = 10.384**, and the test results were all extremely significant.

Except *x*_1_ is not significant to *y*_2_ and *y*_3_, the above test results showed that the established multiple-to-multiple standardized linear regression equations were extremely significant. The path analysis and decision analysis can be performed subsequently.

Secondly, one-to-multiple path analysis of $Y_{\alpha }=\beta_{\alpha }^{*T}x+\varepsilon_{\alpha }\left(\alpha =1,2,\cdots ,p\right)$ was conducted according to the theory before (Method, Part 2.3). The detailed division results of the correlation coefficient and the determination coefficient were listed in **Table 2** and **Table 3**. The decision analysis was also conducted and the results were also listed in **Table 3**.

**Table 2:** The division results of the correlation coefficient about $Y_{\alpha }=\beta_{\alpha }^{*T}x+\varepsilon_{\alpha }\left(\alpha =1,2,3\right)$.

	*x*_*j*_ to *y*_*α*_	Direct effect	*x*_*j*_↔*y*_*α*_	$r_{jk}b_{k\alpha }^{*}$	Indirect effect	$\sum_{j\neq k}r_{kj}b_{j\alpha }^{*}$	Total effect
	*x*_1_ to *y*_1_	0.6767**	*x*_1_↔*x*_2_→*y*_1_	-0.7559	-0.66638(3)	0.2328(1)	0.013(1)
			*x*_1_↔*x*_3_→*y*_1_	0.0921			
1	*x*_2_ to *y*_1_	-1.0631**	*x*_2_↔*x*_1_→*y*_1_	0.4811	0.5860(1)	-0.3115(3)	-0.477(3)
			*x*_2_↔*x*_3_→*y*_1_	0.1049			
	*x*_3_ to *y*_1_	0.251**	*x*_3_↔*x*_1_→*y*_1_	-0.2483	0.1960(2)	0.1970(2)	-0.055(2)
			*x*_3_↔*x*_2_→*y*_1_	0.4444			
	*x*_1_ to *y*_2_	0.1323(3)	*x*_1_↔*x*_2_→*y*_2_	0.2219	0.0927(1)	0.0455(2)	0.225(2)
			*x*_1_↔*x*_3_→*y*_2_	-0.1292			
2	*x*_2_ to *y*_2_	0.3121(2)	*x*_2_↔*x*_1_→*y*_2_	0.0941	-0.0530(2)	0.0914(1)	0.259(1)
			*x*_2_↔*x*_3_→*y*_2_	-0.1471			
	*x*_3_ to *y*_2_	0.3520(1)	*x*_3_↔*x*_1_→*y*_2_	-0.0486	-0.1791(3)	-0.2763(3)	0.173(3)
			*x*_3_↔*x*_2_→*y*_2_	0.1305			
	*x*_1_ to *y*_3_	-0.2150*(3)	*x*_1_↔*x*_2_→*y*_3_	0.4262	0.2432(1)	-0.074(2)	0.028(3)
			*x*_1_↔*x*_3_→*y*_3_	-0.1830			
3	*x*_2_ to *y*_3_	0.5994**(1)	*x*_2_↔*x*_1_→*y*_3_	-0.1529	-0.3613(3)	0.1757(1)	0.238(2)
			*x*_2_↔*x*_3_→*y*_3_	-0.2084			
	*x*_3_ to *y*_3_	0.4986**(2)	*x*_3_↔*x*_1_→*y*_3_	0.0789	-0.1716(2)	-0.3914(3)	0.327(1)
			*x*_3_↔*x*_2_→*y*_3_	-0.2505			

**Table 3:** The division results of determination coefficient $Y_{\alpha }=\beta_{\alpha }^{*T}x+\varepsilon_{\alpha }\left(\alpha =1,2,3\right)$.

	*y*_*α*_←*x*_*j*_→*y*_*α*_	Direct determination	*y*_*α*_←*x*_*j*_↔*x*_*k*_→*y*_*α*_	$r_{jk}b_{k\alpha }^{*}$	indirect determination	Decision coefficient
	*y*_1_←*x*_1_→*y*_1_	0.4579	*y*_1_←*x*_1_↔*x*_2_→*y*_1_	-1.0230	-0.8983	-0.4404**(3)
			*y*_1_←*x*_1_↔*x*_3_→*y*_1_	0.1247		
1	*y*_1_←*x*_2_→*y*_1_	1.1302	*y*_1_←*x*_2_↔*x*_1_→*y*_1_	-1.0230	-1.2461	-0.1159(2)
			*y*_1_←*x*_2_↔*x*_3_→*y*_1_	-0.2231		
	*y*_1_←*x*_3_→*y*_1_	0.0630	*y*_1_←*x*_3_↔*x*_1_→*y*_1_	0.1247	-0.0984	-0.0354(1)
			*y*_1_←*x*_3_↔*x*_2_→*y*_1_	-0.2231		
	*y*_2_←*x*_1_→*y*_2_	0.0175	*y*_2_←*x*_1_↔*x*_2_→*y*_2_	0.0587	0.0245	0.0420*(2)
			*y*_2_←*x*_1_↔*x*_3_→*y*_2_	-0.0342		
2	*y*_2_←*x*_2_→*y*_2_	0.0974	*y*_2_←*x*_2_↔*x*_1_→*y*_2_	0.0587	-0.0331	0.0643**(1)
			*y*_2_←*x*_2_↔*x*_3_→*y*_2_	-0.0918		
			*y*_2_←*x*_3_↔*x*_1_→*y*_2_	-0.0342		
	*y*_2_←*x*_3_→*y*_2_	0.1239			-0.1260	-0.0021(3)
			*y*_2_←*x*_3_↔*x*_2_→*y*_2_	-0.0918		
	*y*_3_←*x*_1_→*y*_3_	0.0462	*y*_3_←*x*_1_↔*x*_2_→*y*_3_	-0.1833	-0.1046	-0.0584(2)
			*y*_3_←*x*_1_↔*x*_3_→*y*_3_	0.0787		
			*y*_3_←*x*_2_↔*x*_1_→*y*_3_	-0.1833		
3	*y*_3_←*x*_2_→*y*_3_	0.3593			-0.4331	-0.0738(3)
			*y*_3_←*x*_2_↔*x*_3_→*y*_3_	-0.2498		
			*y*_3_←*x*_3_↔*x*_1_→*y*_3_	0.0787		
	*y*_3_←*x*_3_→*y*_3_	0.2486			-0.1711	0.0775*(1)
			*y*_3_←*x*_3_↔*x*_2_→*y*_3_	-0.2498		

The t test statistics values of decision coefficient hypothesis testing were listed in **Table 4**.

**Table 4:** t test statistics value of $R_{\alpha \left(j\right)}^{*}$.

	*y*_1_	*y*_2_	*y*_3_
*x*_1_	-3.39774**	2.3205*	-1.2288
*x*_2_	-0.74458	4.5633**	-0.7716
*x*_3_	-0.9793	-0.0636	2.4488*

In one-to-multiple path analysis, the results of correlation coefficient division showed that the total effect of biomass per plant (*x*_1_), single stem grass weight (*x*_2_) and economic coefficient (*x*_3_) are all positive and the largest to panicles per plant (*y*_1_), grain number per pancicle (*y*_2_), 1000-grain weight (*y*_3_), respectively. Differently, the direct effect of *x*_1_ to *y*_1_ is the positive and the largest, while the indirect effect is negative and the smallest. The direct effect of *x*_2_ to *y*_2_, *x*_3_ to *y*_3_ are not the largest, but the total effect becomes the largest through the correlation regulation by the indirect effect. The results of the determination coefficients division and the decision coefficients showed that for *y*_1_, *x*_1_ is a very significant restrictive factor; for *y*_2_, *x*_2_ is a very significant positive factor and *x*_1_ is a significant positive factor; for *y*_3_, *x*_3_ is a significant positive factor. These results meant that single stem grass weight (*x*_2_) and economics coefficient (*x*_3_) need to be increased in order to increase grain number per pancicle (*y*_2_) and 1000-grainweight (*y*_3_), but panicles per plant (*y*_1_) will decrease according due to the negative correlation *x*_2_, *x*_3_ and *y*_1_. Meanwhile, biomass per plant (*x*_1_) should be decreased in order to increase the panicles per plant (*y*_1_), but grain number per pancicle (*y*_2_) will decrease here. The contradictory decision-making results of different independent variables (*x*_*i*_) to different dependent variables (*y*_*i*_) often lead to the confusion of breeders.

Therefore, after the one-to-multiple path analysis, the multiple-to-multiple path analysis was practiced by taking into account the correlation between the dependent variables. According to formula (17–19), the generalized determination coefficient *R*^2^ was divided and the results were listed in **Table 5**.

**Table 5:** The division results about three other types paths of the generalized determination coefficients.

a	*y*_*α*_←*x*_*j*_→*y*_*t*_	direct determination	*y*_*α*_←*x*_*j*_↔*x*_*k*_→*y*_*α*_	$r_{jk}b_{k\alpha }^{*}$
	*y*_1_←*x*_1_→*y*_2_	0.1791	*y*_1_←*x*_1_↔*x*_2_→*y*_2_	0.3003
			*y*_1_←*x*_1_↔*x*_3_→*y*_2_	-0.1748
1	*y*_1_←*x*_2_→*y*_2_	-0.6636	*y*_1_←*x*_2_↔*x*_1_→*y*_2_	-0.2000
			*y*_1_←*x*_2_↔*x*_3_→*y*_2_	0.3128
	*y*_1_←*x*_3_→*y*_2_	-0.1767	*y*_1_←*x*_3_↔*x*_1_→*y*_2_	0.0244
			*y*_1_←*x*_3_↔*x*_2_→*y*_2_	0.0655
	*y*_1_←*x*_1_→*y*_3_	-0.2910	*y*_1_←*x*_1_↔*x*_2_→*y*_3_	0.5768
			*y*_1_←*x*_1_↔*x*_3_→*y*_3_	-0.2477
2	*y*_1_←*x*_2_→*y*_3_	-1.2744	*y*_1_←*x*_2_↔*x*_1_→*y*_3_	0.3253
			*y*_1_←*x*_2_↔*x*_3_→*y*_3_	0.4431
	*y*_1_←*x*_3_→*y*_3_	-0.2503	*y*_1_←*x*_3_↔*x*_1_→*y*_3_	-0.0396
			*y*_1_←*x*_3_↔*x*_2_→*y*_3_	0.1258
	*y*_2_←*x*_1_→*y*_3_	-0.0569	*y*_2_←*x*_1_↔*x*_2_→*y*_3_	0.1128
			*y*_2_←*x*_1_↔*x*_3_→*y*_3_	-0.0484
3	*y*_2_←*x*_2_→*y*_3_	0.3741	*y*_2_←*x*_2_↔*x*_1_→*y*_3_	-0.0955
			*y*_2_←*x*_2_↔*x*_3_→*y*_3_	-0.1301
	*y*_2_←*x*_3_→*y*_3_	0.3510	*y*_2_←*x*_3_↔*x*_1_→*y*_3_	0.0556
			*y*_2_←*x*_3_↔*x*_2_→*y*_3_	-0.1764

The specific calculation of path vector structure is as follows:

$$\begin{array}{l} R^{2}\approx tr\left(B\right)=\left(1.2883,1.1030,1.2086\right)\left[\begin{array}{l} 0.5297\\ 0.1717\\ 0.2997 \end{array}\right]\\ \;+\left(0.3583,0.5142,0.2012\right)\left[\begin{array}{l} -0.3331\\ -0.6323\\ 0.3861 \end{array}\right]=0.8670 \end{array}$$ (23)

From the previous calculation, we can get *tr*(*B*) = 0.8671. The above division of the generalized coefficient of determination is reasonable according to *R*^2^≈*tr*(*B*). The decision analysis of the model was carried out continually. The decision coefficient of each independent variable to *Y* = (*y*_1_,*y*_2_,*y*_3_)^*T*^ was calculated as follows:

$$\begin{array}{l} R_{y\left(1\right)}=\left(\theta_{11},\theta_{22},\theta_{33}\right)\times \left(\underset{\underset{3\times 1}{y}\leftarrow x_{1}\to \underset{3\times 1}{y}}{\left[\begin{array}{l} R_{1\left(1\right)}^{2}\\ R_{1\left(2\right)}^{2}\\ R_{1\left(3\right)}^{2} \end{array}\right]}+\underset{\underset{3\times 1}{y}\leftarrow x_{1}↔x_{2}\to \underset{3\times 1}{y}}{\left[\begin{array}{l} R_{12\left(1\right)}\\ R_{12\left(2\right)}\\ R_{12\left(3\right)} \end{array}\right]}+\underset{\underset{3\times 1}{y}\leftarrow x_{1}↔x_{3}\to \underset{3\times 1}{y}}{\left[\begin{array}{l} R_{13\left(1\right)}\\ R_{13\left(2\right)}\\ R_{13\left(3\right)} \end{array}\right]}\right)\\ \;+\left(\theta_{12},\theta_{13},\theta_{23}\right)\left(\underset{\underset{\left(\alpha <t\right)}{y_{\alpha }\leftarrow x_{1}\to y_{t}}}{\left[\begin{array}{l} R_{1\left(12\right)}\\ R_{1\left(13\right)}\\ R_{1\left(23\right)} \end{array}\right]}+\underset{y_{\alpha }\leftarrow \left(\begin{array}{l} x_{1}↔x_{2}\\ x_{2}↔x_{1} \end{array}\right)\to y_{t}}{\left[\begin{array}{l} R_{12\left(12\right)}+R_{21\left(12\right)}\\ R_{12\left(13\right)}+R_{21\left(13\right)}\\ R_{12\left(23\right)}+R_{21\left(23\right)} \end{array}\right]}+\underset{y_{\alpha }\leftarrow \left(\begin{array}{l} x_{1}↔x_{3}\\ x_{3}↔x_{1} \end{array}\right)\to y_{t}}{\left[\begin{array}{l} R_{13\left(12\right)}+R_{31\left(12\right)}\\ R_{13\left(13\right)}+R_{31\left(13\right)}\\ R_{13\left(23\right)}+R_{31\left(23\right)} \end{array}\right]}\right)\\ \;=‐0.5916+0.2060=‐0.3856^{**} \end{array}$$ (24)

Similar available: *R*_*y*(2)_ = −0.1157, *R*_*y*(3)_ = 0.0906*. According to the decision coefficient, the t test about *R*_*y*(*j*)_ is further conducted, and the result is *t*_1_ = −4.3943**, *t*_2_ = 0.9293, *t*_3_ = 2.0785**. In addition, it should be noted that the determination coefficients of *x*_*j*_ and *x*_*j*_↔*x*_*k*_ to *y*_*α*_ have been calculated by one-to-multiple path analysis model (Table 3). The comparison of the results of Table 3 and those of Table 5 demonstrated that great changes have taken place in the regulation of *x*_*j*_ to *Y* when the correlation among dependent variables was considered. Firstly, the direct and indirect regulations of *x*_*j*_, *x*_*j*_↔*x*_*k*_ to *Y* also were greatly affected by the correlation among *Y* because of common *X*. As shown in Table 3, the direct determination of *x*_2_ to *y*_1_, *y*_2_ were both positive, respectively ($R_{1\left(2\right)}^{2}=1.1302y_{1}\leftarrow x_{2}\to y_{1};R_{2\left(2\right)}^{2}=0.0974$ *y*_2_←*x*_2_→*y*_2_). But in Table 5, the direct determination of *x*_2_ to *y*_1_ and *y*_2_ became negative (*R*_12(2)_ = -0.6636 *y*_1_←*x*_2_→*y*_2_). This change was due to the consideration of the negative and large correlation of *y*_1_ and *y*_2_ $\left(r_{y_{1}y_{2}}=-0.255\right)$. Similarly, the direct determination of *x*_2_ to *y*_2_ and *y*_3_ was still changed (*R*_23(2)_ = 0.3741 *y*_2_←*x*_2_→*y*_3_), compared to the previous determination coefficient $(R_{2\left(2\right)}^{2}=0.0974\;y_{2}\leftarrow x_{2}\to y_{2};\;R_{3\left(2\right)}^{2}=0.3593\;y_{3}\leftarrow x_{2}\to y_{3})$. Different from the above, this change was small and both were positive. This phenomenon showed that the small correlation of *y*_2_ and *y*_3_ $\left(r_{y_{2}y_{3}}=-0.058\right)$ had little influence on the direct determination of *x*_2_ to *y*_2_ and *y*_3_. The direct determination of *x*_3_ to *y*_2_ and *y*_3_ was exactly like the direct determination of *x*_2_ to *y*_2_ and *y*_3_. The indirect determination due to the correlation of *x*_*j*_↔*x*_*k*_ also changed a lot because of consideration of the correlation among *Y*. For example, the indirect determination of *x*_1_↔*x*_2_ to *y*_1_and *y*_3_ was 0.5768(*y*_1_←*x*_1_↔*x*_2_→*y*_3_) and 0.3253(*y*_1_←*x*_2_↔*x*_1_→*y*_3_). It’s strange that the original indirect determination of *x*_1_↔*x*_2_to *y*_1_, *x*_1_↔*x*_2_ to *y*_3_ were -1.023 (*y*_1_←*x*_1_↔*x*_2_→*y*_1_) and 0.1833(*y*_3_←*x*_1_↔*x*_2_→*y*_3_), respectively. It is obvious that the strong negative correlation of *y*_1_ and *y*_3_ $\left(r_{y_{1}y_{3}}=-0.383\right)$ led to the change of indirect regulation. These big changes were enough to show the importance of considering the correlation among *Y*. There were similar changes in the direct determination of *x*_1_↔*x*_2_ to *y*_1_and *y*_2_(*y*_1_←*x*_1_↔*x*_2_→*y*_2_, *y*_1_←*x*_2_↔*x*_1_→*y*_2_) and *x*_2_↔*x*_3_ to *y*_1_ and *y*_3_(*y*_1_←*x*_2_↔*x*_3_→*y*_3_ *y*_1_←*x*_3_↔*x*_2_→*y*_3_). Secondly, the decision coefficients results showed that *x*_1_ is the very significant restrictive decision factor of *Y* = (*y*_1_,*y*_2_,*y*_3_)^*T*^ (*R*_*y*(1)_ = −0.3856**). But *x*_1_ is the significant positive decision factor to *y*_2_(*R*_*y*2(1)_ = 0.0420*) and is not significant to *y*_3_(*R*_*y*3(1)_ = -0.0584). This phenomena seemed to be caused by the very significant negative decision making effect of *x*_1_ to *y*_1_, and strong negative correlation between *y*_1_ and *y*_2_ $\left(r_{y_{1}y_{2}}=-0.255\right)$, y_1_ and *y*_3_ $\left(r_{y_{1}y_{3}}=-0.383\right)$. For *x*_2_, there is no point in making a decision. (*R*_*y*(2)_ = −0.1157). And *x*_3_ became a significant positive decision factor to $Y={\left(y_{1},y_{2},y_{3}\right)}^{T}\left(R_{y_{\left(3\right)}}=0.0906^{*}\right)$. However, *x*_3_ is significant only to *y*_3_ $\left(R_{y_{3}\left(3\right)}=0.0775^{*}\right)$, and is not significant to *y*_1_, *y*_2_ in one-to-multiple path analysis. Obviously, the correlation among *Y* due to common *X* makes a big difference in the decision making. The results showed that the economic coefficient (*x*_3_) should be increased, the biomass per plant (*x*_1_) should be appropriately reduced and the single stem grass weight (*x*_2_) should remain unchanged in the process of wheat breeding. These results were in accordance with the existing documents results [28]. In short, the consideration of the correlation among *Y* caused a big change of the direct determination, the indirect determination and the decision analysis results of *x*_*j*_ to *Y*. And the greater the correlation among *Y* is, the greater the impact on regulation.

## 4 Discussion

In this article, the multiple-to-multiple path analysis model was proposed based on multivariate linear regression analysis, which can be regarded as a generalization of one-to-multiple path analysis model based on univariate linear regression analysis. The innovation of this model is the multiple-to-multiple path analysis central theorem. The correlation among *Y* caused by common *X* was considered in the system analysis including multiple independent variables and multiple dependent variables. As Fig 2 shown, the other three types of paths (*y*_*α*_←*x*_*j*_→y_*t*_, *y*_*α*_←*x*_*j*_↔*x*_*k*_→*y*_*t*_, *y*_*α*_←*x*_*k*_↔*x*_*j*_→*y*_*t*_) generated in multiple-to-multiple path analysis model besides the two types of paths (*y*_*α*_←*x*_*j*_→*y*_*α*_, *y*_*α*_←*x*_*j*_↔*x*_*k*_→*y*_*α*_) in one-to-multiple path analysis. Along these five types of paths, the generalized determination coefficient *R*^2^ was divided into the direct determination and the indirect determination according to *R*^2^≈*tr*(*B*). This division can clearly show the complex regulatory mechanisms among variables. Still further, the generalized decision coefficient *R*_*y*(*j*)_ was constructed by synthesizing all the items related to *x*_*j*_, which was used to express the comprehensive decision-making ability of *x*_*j*_ to *Y* = (*Y*_1_,*Y*_2_,⋯,*Y*_*p*_)^*T*^. In fact, the direct and indirect determinations all were products of corresponding path coefficients. The quantitative expression of the regulation among variables is helpful for decision makers to make more reasonable and optimized decision suggestions for target variables. The analysis results of the wheat data in arid areas strongly confirm this. It is worth mentioning that the path analysis of any closed system can be made according to the multiple-to-multiple path analysis central theorem. However, the application of multiple-to-multiple path analysis model still has some limitations. Firstly, the model is only applicable to the causal relationship analysis among multiple dependent variables and independent variables with correlation. Secondly, the difference between the generalized determination *R*^2^ and *tr*(*B*) is relatively large when the correlation among variables is very strong in multiple-to-multiple linear regression analysis, that is, the value of the correlation coefficient in correlation matrix is almost 1. Here, the division of the generalized determination coefficient *R*^2^ based on *R*^2^≈*tr*(*B*) is very different from the actual result. Therefore, other division methods need to be further considered.

## 5 Conclusion

In the multiple-to-multiple path analysis model, the correlation among dependent variables caused by common independent variable is considered, besides the correlation among independent variables. Taking into account more correlation information analysis makes the results more practical and instructive.

## References

[1] Naes T, Romano R, Tomic O, et al. Sequential and orthogonalized PLS (SO-PLS) regression for path analysis: Order of blocks and relations between effects. J Chemom. 2020; e3243.

[2] Wright S. On the nature of size factors. Genetics. 1918; 3(4): 367–374. 17245910 10.1093/genetics/3.4.367 PMC1200442

[3] Wright S. Correlation and causation. J Agric Res. 1921; 20: 557–585.

[4] Bollen KA. Structural Equations with Latent Variables. NY: Wiley. 1989.

[5] Duncan OD. Path analysis: sociological examples. Am J Sociol. 1966; 72(1): 1–16.

[6] Finney JM. Indirect effects in path analysis. Socio Meth Res. 1972; 1(2): 175–186.

[7] Greene VL. An algorithm for total and indirect causal effects. Polit Anal. 1977; 369–381.

[8] Berg PVD, Arentze T, Timmermans H. A path analysis of social networks, telecommunication and social activity-travel patterns. Transp Res Part C. Emerg Technol. 2013; 26: 256–268.

[9] Kang DH. An path analysis of the elderly’s deprivation experience on the thinking of suicide. J Soc Sci. 2019; 58: 197–245.

[10] Hwang HJ, Chun HY, Ok KH. The path analysis of parental divorce on children’s emotional and behavioural problems: Through child-rearing behaviours and children’s self-esteem. J Korean Home Econ Assoc. 2010; 48(7): 99–110.

[11] Diao ZJ, Chen B. Correlation and path coefficient analysis between thermal extraction yield and coal properties. Energy Sources Part A. Recovery Util Environ Eff. 2016; 38(22): 3412–3416.

[12] Cankaya S, Abaci SH. Path analysis for determination of relationships between some body measurements and live weight of German fawn x hair crossbred kids. Kafkas Univ Vet Fak Derg. 2012; 18 (5): 769–773. 10.9775/kvfd.2012.6376

[13] Norris D, Brown D, Moela AK, et al. Path coefficient and path analysis of body weight and biometric traits in indigenous goats. Indian J Anim Res. 2015; 49 (5): 573–578.

[14] Marjanović-Jeromela A, Marinković R, Mijić A, et al. Correlation and path analysis of quantitative traits in winter rapeseed (brassica napus l.). Agric Conspec Sci. 2008; 73: 13–18.

[15] Barbosa RP, Alcntara-Neto F, Gravina LM, et al. Early selection of sugarcane using path analysis. Genet Mol Res. 2017; 16(1): gmr16019038. 10.4238/gmr16019038 28198498

[16] Grace JB, Pugesek BH. On the use of path analysis and related procedures for the investigation of ecological problems. Am Nat. 1998; 152 (1):151–159. 10.1086/286156 18811408

[17] Kunanitthaworn N, Wongpakaran T, Wongpakaran N, et al. Factors associated with motivation in medical education: a path analysis. BMC Med Educ. 2018; 18: 140. 10.1186/s12909-018-1256-5 29914462 PMC6006981

[18] Costello RM. Premorbid social competence construct generalizability across ethnic groups: Path analyses with two premorbid social competence components. J Consult Clin Psychol. 1978; 46(5): 1164–1165. 10.1037//0022-006x.46.5.1164 701557

[19] Jöreskog KG. Structural analysis of covariance and correlation matrices. Psychometrika. 1978; 43(4): 443–477.

[20] Graff J, Schmidt P. A general model for decomposition of effects. North–Holl Publ Co. 1982; 131–148. Netherlands.

[21] Yuan ZF, Zhou JY, Guo MC, et al. Decision coefficients-decision indicators in path analysis. J Northwest A&F Univ (Nat Sci Ed). 2001; 29(5): 131–133. China.

[22] Xie XL, Yuan ZF. Statistical test of decision coefficient and its application in breeding. J Northwest A&F Univ (Nat Sci Ed). 2013; 41(3): 111–114.China.

[23] Mei Y, Guo W, Fan S, et al. Analysis of decision-making coefficients of the lint yield of upland cotton (Gossypium hirsutum L.). Euphytica. 2014; 196(1): 95–104.

[24] Du JL, Li ML, Yuan ZF, et al. A decision analysis model for KEGG pathway analysis. BMC Bioinform. 2016; 17(1): 407. 10.1186/s12859-016-1285-1 27716040 PMC5053338

[25] Du JL, Yuan ZF, Ma ZW, et al. KEGG-PATH: Kyoto encyclopedia of genes and genomes-based pathway analysis using a path analysis model. Mol Biosyst. 2014; 10(9): 2441–2447. 10.1039/c4mb00287c 24994036

[26] Xie XL, Du JL, Xie XZ, et al. Generalized complex correlation coefficient and its application in wheat breeding. J Triticeae Crop. 2017; 37(1): 87–93. China.

[27] Duleba AJ, Olive DL. Regression analysis and multivariate analysis. Semin Reprod Endocrinol. 1996; 14(2): 139–153. 10.1055/s-2007-1016322 8796937

[28] Zhang Z, Wang D. Wheat drought-resistant ecological breeding. Xi’an: Shaanxi People’s Education Press; 1992. China.

